# Supplementary material for: New N-Adducts of Thiadiazole and Thiazoline with Levoglucosenone and Evaluation of Their Significant Cytotoxic (Anti-Cancer) Activity
Source: Cancers (Basel). 2024 Jan 2;16(1):216. doi: 10.3390/cancers16010216 (PMC10777969; doi:10.3390/cancers16010216)
Supplement: Supplementary file 1 [file cancers-16-00216-s001.zip › cancers-2784749-supplementary.pdf]

Supporting Information for

New *N*-adducts of thiadiazole and thiazoline with levoglucosenone and their evaluation of significant cytotoxic (anticancer) activity

Tomasz Poplawski[1], Grzegorz Galita[2], Joanna Sarnik[3], Anna Macieja[1], Roman Bielski[4], Donald E. Mencer[5] Zbigniew J. Witczak[4*]

[1] *Department of Pharmaceutical Microbiology and Biochemistry, Medical University, 92-215 Lodz, Poland*
[2] *Department of Clinical Chemistry and Biochemistry, Medical University, 92-215 Lodz, Poland*
[3] *Department of Rheumatology, Medical University, 90-050 Lodz, Poland.*
[4] *Department of Pharmaceutical Sciences, Nesbitt School of Pharmacy, Wilkes University, 84 W. South Street, Wilkes-Barre, PA 18766, USA*
[5] *Department of Chemistry and Biochemistry, Wilkes University, 84 W. South Street, Wilkes-Barre, PA 18766, USA*

Email: zbigniew.witczak@wilkes.edu.

Contents

1. Synthetic procedures and data characterization
2. Molecular properties of *FCP23 and FCP26*
3. X-Ray single crystal analysis and ORTEP for FCP26
4. ^{1}H, ^{13}C NMR spectra *FCP23 and FCP26*
5. MS spectra *FCP23*
6. The cytotoxicity of FCP23 and FCP26

1. General information

All reagents and solvents were used as purchased without further purification. Unless otherwise stated, all reactions were carried out under inert atmosphere in oven-dried glassware with dried solvents. All solvents were dried and degassed by standard methods before use. Optical rotation was measured using a JASCO P-2000 Digital Polarimeter.
^{1}H NMR and ^{13}C NMR spectra were recorded on 400MHz Bruker Avance. 2D experiments (COSY and HSQC) were performed to enhance assignments. Chemical shifts (δ-scale) are reported in ppm with TMS (0 ppm) as internal standard for ^{1}H NMR and the residual solvent signals ($CDCl_3$: 7.26, for ^{1}H NMR and ($CDCl_3$: 77.0 ppm) for ^{13}C NMR.
Thin layer chromatography was performed on silica gel coated TLC plates and visualized under UV light (at 254nm); detection was executed by exposing to iodine (I_2) vapor. Mass spectra were obtained using APCI-Quadrupole technique. The melting points (mp) were obtained on an ElectroThermal FARGO MP-2D capillary melting point apparatus and were uncorrected. Chemical names were generated by ChemDraw Professional V.15.1.0.144 software.

2. Synthetic procedures and characterization data.

General procedure for the synthesis of FCP23 and FCP 26

To a solution of levoglucosenone (**1**) 1.28g (0.01mole) in 35 mL of chloroform a 0.01 mole of thiols **2-3** was added and magnetically stirred for 5.min. After that time, 1 mL of triethylamine was added drop-wise and the solution was refluxed for 24-48hr. Upon overnight cooling at 5°C the crystalline residue was filtered off and recrystallized from ethanol. The crystalline products were filtered off and dried on fresh air.

(1*S*,2*S*,5*R*)-2-(3-amino-5-thioxo-1,2,4-thiadiazol-4(5*H*)-yl)-6,8-dioxabicyclo[3.2.1]octan-4-one (23)

Yield (1.9 g, 73%), White solid crystals; m.p.: 137–139 °C; *R* = 0.74 (1:4, hexane–EtOAc); $[\alpha]^{30}$– 124.3 (*c* 0.81, CHCl); ^{1}H-NMR: δ (400 MHz; $CDCl_3$), 5.37 (d, 1H, H-1), 5.76 (d, $J_{3a\text{-}4}$ = 9.6 Hz, 1H, H-4), 2.54 (d, 1H, H-3e), 4.92 (*d*, $J_{5\text{-}6exo}$ = 5.5 Hz, 1H, H-5), 4.23 (d, J_{gem} = 8.2 Hz, 1H, H-6*endo*), 4.04 (dd, J_{gem} = 8.2 Hz, $J_{5\text{-}6exo}$ = 5.5 Hz, 1H, H-6*exo*), 3.12 (dd, J_{gem} = 9.6 Hz, 1H, H-3a), 2.83 (d, J_{gem} =18.6 Hz, 1H, H-3b), 9.15 (s, 1H, -NH_2); ^{13}C-NMR δ (100 MHz; $CDCl_3$) 31.03 (C-3), 48.04 (C-6), 56.51 (C-4), 66.41, 73.81 (C-5), 92.58, 102.86 (C-1), 158. 95, 177.52 (C-2, C=O). HRMS *m/z* [M + H]: Calcd for $C_8H_9N_3O_3S_2$: 259.30. Found: 260.00. For copies of the ^{1}H-NMR and ^{13}C-NMR see Supplementary Materials.

(1*S*,2*S*,5*R*)-2-(2-thioxothiazolidin-3-yl)-6,8-dioxabicyclo[3.2.1]octan-4-one (26)

Yield (1.82 g, 74%), White solid crystals; m.p.: 144–146 °C; *R* = 0.57 (1:1, hexane–EtOAc); $[\alpha]^{30}$– 134.2 (*c* 0.84, CHCl); ^{1}H-NMR: δ (400 MHz; $CDCl_3$), 5.31 (d, 1H, H-1), 5.72 (d, $J_{3a\text{-}4}$ = 9.4 Hz, 1H, H-4), 2.58 (d, 1H, H-3e), 4.92 (d, $J_{5\text{-}6exo}$ = 5.3 Hz, 1H, H-5), 4.23 (d, J_{gem} = 8.4 Hz, 1H, H-6*endo*), 4.04 (dd, J_{gem} = 8.4 Hz, $J_{5\text{-}6exo}$ = 5.5 Hz, 1H, H-6*exo*) 3.16 (dd, J_{gem} = 9.4 Hz, 1H, H-3a), 3.06 (d, J_{gem} = 16.9 Hz, 2H, C-4 thiazoline) 3.21(d, J_{gem} = 17.1 Hz, 2H, C-5, thiazoline), 2.81 (d, J_{gem} =18.4 Hz, 1H, H-3b); ^{13}C-NMR δ (100 MHz; $CDCl_3$) 31.1 (C-3), 36.7 (C-3, CH_2-thiazoline), 39.4 (C-4, CH_2-thiazoline), 68.4 (C-6), 70.6 (C-4), 78.2 (C-5), 104.4 (C-1), 128.7 (C-2, thiazoline), 207.2 (C-2, C=O). HRMS *m/z*, [M + H]: Calcd for $C_9H_{11}NO_3S_2$: 245.02, Found: 246.0252. For copies of the ^{1} H-NMR and ^{13} C-NMR see Supplementary Materials.

Crystal Structure Report for 26

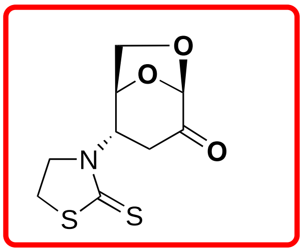

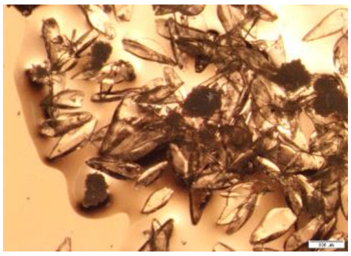

(1*S*,2*S*,5*R*)-2-(2-thioxothiazolidin-3-yl)-6,8-dioxabicyclo[3.2.1]octan-4-one

A specimen of $C_9H_{11}NO_3S_2$ was used for the X-ray crystallographic analysis. The X-ray intensity data were measured (λ = 0.71073 Å).

The total exposure time was 16.27 hours. The frames were integrated with the Bruker SAINT software package using a narrow-frame algorithm. The integration of the data using an orthorhombic unit cell yielded a total of 17954 reflections to a maximum θ angle of 33.17° (0.65 Å resolution), of which 3803 were independent (average redundancy 4.721, completeness = 96.0%, R_{int} = 2.41%, R_{sig} = 1.92%) and 3608 (94.87%) were greater than 2σ(F^2). The final cell constants of <u>a</u> = 7.6265(6) Å, <u>b</u> = 8.5404(6) Å, <u>c</u> = 16.1668(12) Å, volume = 1053.00(14) Å^3, are based upon the refinement of the XYZ-centroids of 9879 reflections above 20 σ(I) with 5.039° < 2θ < 65.80°. Data were corrected for absorption effects using the Multi-Scan method (SADABS). The ratio of minimum to maximum apparent transmission was 0.776.

The structure was solved and refined using the Bruker SHELXTL Software Package, using the space group $P2_12_12_1$, with Z = 4 for the formula unit, $C_9H_{11}NO_3S_2$. The final anisotropic full-matrix least-squares refinement on F^2 with 180 variables converged at R1 = 3.90%, for the observed data and wR2 = 9.34% for all data. The goodness-of-fit was 1.141. The largest peak in the final difference electron density synthesis was 0.516 e^-/Å^3 and the largest hole was -0.321 e^-/Å^3 with an RMS deviation of 0.069 e^-/Å^3. On the basis of the final model, the calculated density was 1.547 g/cm^3 and F(000), 512 e^-.

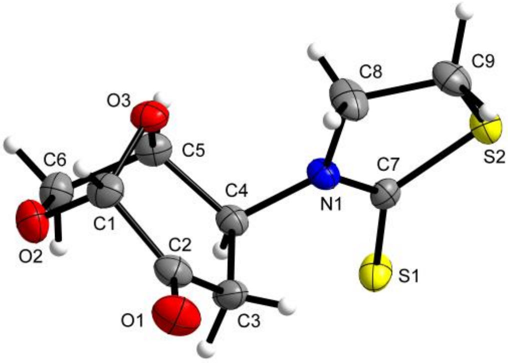

Table S1. Sample and crystal data for 26

**Identification code**	ZJW26	
**Chemical formula**	$C_9H_{11}NO_3S_2$	
**Formula weight**	245.31 g/mol	
**Temperature**	150(2) K	
**Wavelength**	0.71073 Å	
**Crystal system**	orthorhombic	
**Space group**	P $2_12_12_1$	
**Unit cell dimensions**	a = 7.6265(6) Å	α = 90°
	b = 8.5404(6) Å	β = 90°
	c = 16.1668(12) Å	γ = 90°
**Volume**	1053.00(14) $Å^3$	
**Z**	4	
**Density (calculated)**	1.547 g/cm^3	
**Absorption coefficient**	0.491 mm^{-1}	
**F(000)**	512	

Table S2. Data collection and structure refinement for 26

**Theta range for data collection**	2.52 to 33.17°	
**Index ranges**	-11<=h<=11, -12<=k<=12, -23<=l<=24	
**Reflections collected**	17954	
**Independent reflections**	3803 [R(int) = 0.0241]	
**Coverage of independent reflections**	96.0%	
**Absorption correction**	Multi-Scan	
**Structure solution technique**	Intrinsic phasing	
**Structure solution program**	SHELXT 2014/5 (Sheldrick, 2014)	
**Refinement method**	Full-matrix least-squares on F^2	
**Refinement program**	SHELXL-2018/3 (Sheldrick, 2018)	
**Function minimized**	$\Sigma w(F_o^2 - F_c^2)^2$	
**Data / restraints / parameters**	3803 / 0 / 180	
**Goodness-of-fit on F^2**	1.141	
**Δ/σ_{max}**	0.001	
**Final R indices**	3608 data; $I>2\sigma(I)$	R1 = 0.0390, wR2 = 0.0909
	all data	R1 = 0.0428, wR2 = 0.0934
**Weighting scheme**	$w=1/[\sigma^2(F_o^2)+(0.0331P)^2+0.7006P]$ where $P=(F_o^2+2F_c^2)/3$	
**Absolute structure parameter**	0.014(19)	
**Largest diff. peak and hole**	0.516 and -0.321 eÅ^{-3}	
**R.M.S. deviation from mean**	0.069 eÅ^{-3}	

Table S3. Atomic coordinates and equivalent isotropic atomic displacement parameters (Å^2) for 26

U(eq) is defined as one third of the trace of the orthogonalized U_{ij} tensor.

	x/a	y/b	z/c	U(eq)
S1	0.49069(10)	0.10475(7)	0.47054(4)	0.02836(14)
S2	0.44453(8)	0.26515(7)	0.63164(4)	0.02556(13)
O1	0.1833(3)	0.7305(3)	0.31897(14)	0.0391(5)
O2	0.5911(3)	0.7136(3)	0.27465(13)	0.0344(4)
O3	0.5845(2)	0.7279(2)	0.41466(12)	0.0271(4)
N1	0.4787(3)	0.4172(2)	0.49556(12)	0.0217(4)
C1	0.4889(4)	0.7643(3)	0.34276(16)	0.0281(5)
C2	0.3189(4)	0.6708(3)	0.34139(16)	0.0268(5)
C3	0.3352(4)	0.5006(3)	0.36439(18)	0.0261(5)
C4	0.5077(4)	0.4515(3)	0.40734(13)	0.0218(4)
C5	0.6520(3)	0.5743(3)	0.39613(16)	0.0253(5)
C6	0.7087(4)	0.5940(4)	0.30609(18)	0.0312(5)
C7	0.4725(3)	0.2711(2)	0.52426(13)	0.0194(4)
C8	0.4637(5)	0.5431(3)	0.55712(17)	0.0353(6)
C9	0.4014(4)	0.4734(3)	0.63817(17)	0.0295(5)

Table S4. Bond lengths (Å) for 26

S1-C7	1.671(2)	S2-C7	1.750(2)
S2-C9	1.812(3)	O1-C2	1.209(3)
O2-C1	1.418(3)	O2-C6	1.451(4)
O3-C1	1.407(3)	O3-C5	1.441(3)
N1-C7	1.332(3)	N1-C8	1.469(3)
N1-C4	1.473(3)	C1-C2	1.523(4)
C1-H1	0.97(3)	C2-C3	1.505(4)
C3-C4	1.546(4)	C3-H3B	0.93(5)
C3-H3A	0.89(4)	C4-C5	1.531(4)
C4-H4	0.94(3)	C5-C6	1.528(4)
C5-H5	0.90(4)	C6-H6B	0.98(4)
C6-H6A	0.97(4)	C8-C9	1.515(4)
C8-H8B	0.99(4)	C8-H8A	0.89(4)
C9-H9B	0.98(4)	C9-H9A	0.99(4)

Table S5. Bond angles (°) for 26

C7-S2-C9	92.95(12)	C1-O2-C6	106.4(2)
C1-O3-C5	102.39(18)	C7-N1-C8	116.5(2)
C7-N1-C4	121.92(18)	C8-N1-C4	121.48(19)
O3-C1-O2	106.8(2)	O3-C1-C2	109.7(2)
O2-C1-C2	107.3(2)	O3-C1-H1	109.(2)
O2-C1-H1	112.(2)	C2-C1-H1	112.(2)
O1-C2-C3	123.5(3)	O1-C2-C1	120.7(3)
C3-C2-C1	115.6(2)	C2-C3-C4	116.3(2)
C2-C3-H3B	107.(3)	C4-C3-H3B	108.(3)
C2-C3-H3A	105.(2)	C4-C3-H3A	111.(2)
H3B-C3-H3A	109.(4)	N1-C4-C5	111.03(19)
N1-C4-C3	111.2(2)	C5-C4-C3	111.89(19)
N1-C4-H4	108.(2)	C5-C4-H4	106.3(19)
C3-C4-H4	108.(2)	O3-C5-C6	101.5(2)
O3-C5-C4	110.0(2)	C6-C5-C4	113.1(2)
O3-C5-H5	109.(2)	C6-C5-H5	112.(2)
C4-C5-H5	111.(2)	O2-C6-C5	103.7(2)
O2-C6-H6B	110.(2)	C5-C6-H6B	111.(2)
O2-C6-H6A	108.(2)	C5-C6-H6A	110.(2)
H6B-C6-H6A	114.(3)	N1-C7-S1	127.77(17)
N1-C7-S2	112.14(16)	S1-C7-S2	120.08(13)
N1-C8-C9	108.8(2)	N1-C8-H8B	109.(3)
C9-C8-H8B	113.(3)	N1-C8-H8A	103.(2)
C9-C8-H8A	109.(2)	H8B-C8-H8A	115.(3)
C8-C9-S2	106.14(18)	C8-C9-H9B	110.(3)
S2-C9-H9B	107.(3)	C8-C9-H9A	112.(3)
S2-C9-H9A	108.(3)	H9B-C9-H9A	113.(4)

Table S6. Anisotropic atomic displacement parameters (Å²) for 26

The anisotropic atomic displacement factor exponent takes the form: $-2\pi^2[h^2 a^{*2} U_{11} + ... + 2 h k a^* b^* U_{12}]$

	U_{11}	U_{22}	U_{33}	U_{23}	U_{13}	U_{12}
S1	0.0365(3)	0.0188(2)	0.0298(3)	-0.0038(2)	0.0094(3)	-0.0026(2)
S2	0.0286(3)	0.0258(3)	0.0223(2)	0.0014(2)	0.0027(2)	0.0029(2)
O1	0.0350(10)	0.0422(12)	0.0401(11)	0.0002(10)	0.0097(9)	0.0120(10)
O2	0.0411(11)	0.0337(10)	0.0283(9)	0.0083(8)	0.0034(8)	0.0005(9)
O3	0.0321(9)	0.0204(7)	0.0287(8)	0.0021(7)	0.0053(7)	0.0023(7)
N1	0.0269(10)	0.0179(7)	0.0202(8)	0.0029(6)	0.0007(7)	0.0028(7)
C1	0.0349(12)	0.0180(9)	0.0313(11)	0.0012(8)	0.0030(10)	0.0006(9)
C2	0.0282(11)	0.0284(11)	0.0239(10)	0.0047(9)	0.0023(9)	0.0051(9)
C3	0.0263(11)	0.0258(11)	0.0262(11)	0.0002(9)	0.0053(10)	0.0046(9)
C4	0.0264(10)	0.0187(8)	0.0202(9)	0.0016(7)	0.0003(8)	0.0017(8)
C5	0.0234(11)	0.0241(11)	0.0284(11)	0.0004(9)	0.0019(9)	0.0030(9)
C6	0.0292(13)	0.0320(13)	0.0325(13)	0.0016(11)	0.0052(10)	0.0007(11)
C7	0.0164(8)	0.0189(8)	0.0230(9)	0.0010(7)	0.0014(7)	0.0013(7)
C8	0.057(2)	0.0227(11)	0.0261(11)	-0.0068(9)	0.0070(12)	0.0026(12)
C9	0.0379(13)	0.0276(11)	0.0230(11)	-0.0063(9)	0.0005(10)	0.0056(10)

Table S7. Hydrogen atomic coordinates and isotropic atomic displacement parameters (Å²) for 26

	**x/a**	**y/b**	**z/c**	**U(eq)**
H1	0.468(5)	0.876(4)	0.341(2)	0.032(9)
H3B	0.325(6)	0.443(6)	0.316(3)	0.054(13)
H3A	0.243(5)	0.481(4)	0.396(2)	0.028(9)
H4	0.549(4)	0.360(4)	0.382(2)	0.023(8)
H5	0.743(5)	0.555(4)	0.430(2)	0.027(8)
H6B	0.691(5)	0.497(5)	0.275(2)	0.038(11)
H6A	0.827(5)	0.635(5)	0.304(2)	0.035(10)
H8B	0.383(6)	0.624(5)	0.535(3)	0.046(11)
H8A	0.574(5)	0.574(5)	0.563(2)	0.033(10)
H9B	0.274(6)	0.486(5)	0.644(3)	0.049(12)
H9A	0.467(6)	0.515(5)	0.686(3)	0.054(13)

ZJW23 ~19 mg in ~0.67 mL DMSO
hemiketal

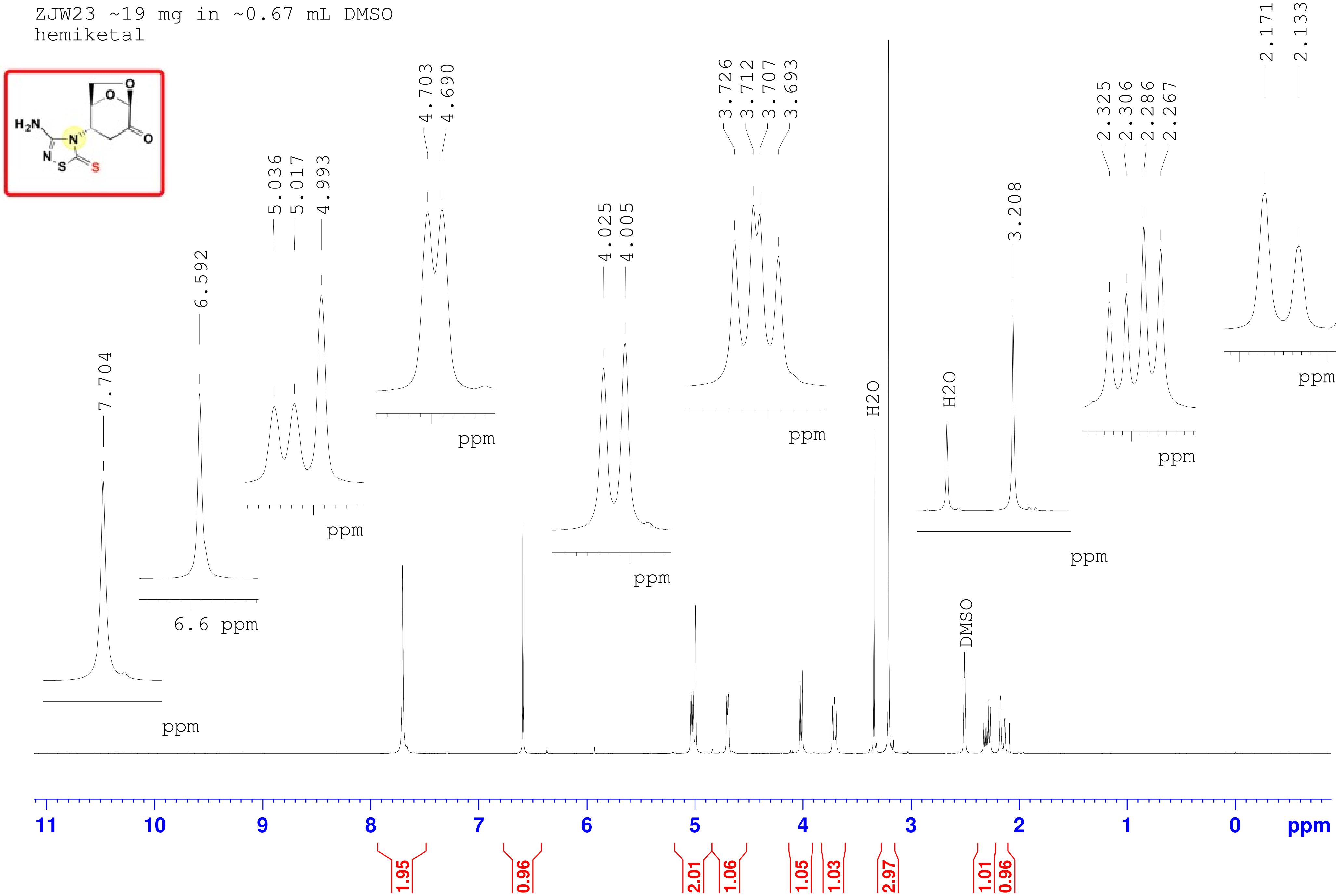

ZJW-23 ~18 mg in ~0.67 mL DMSO
hemiketal

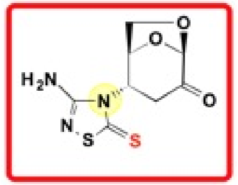

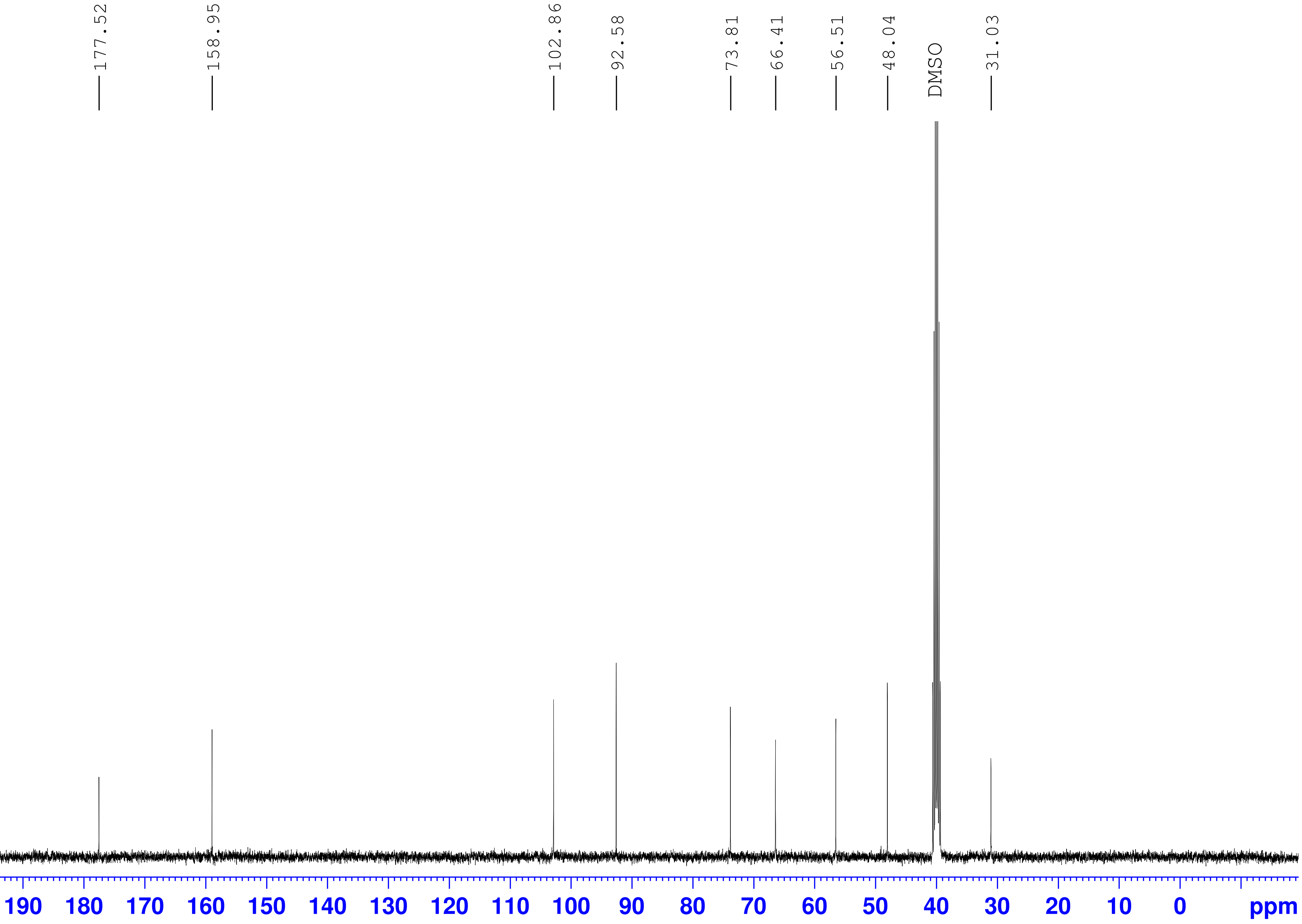

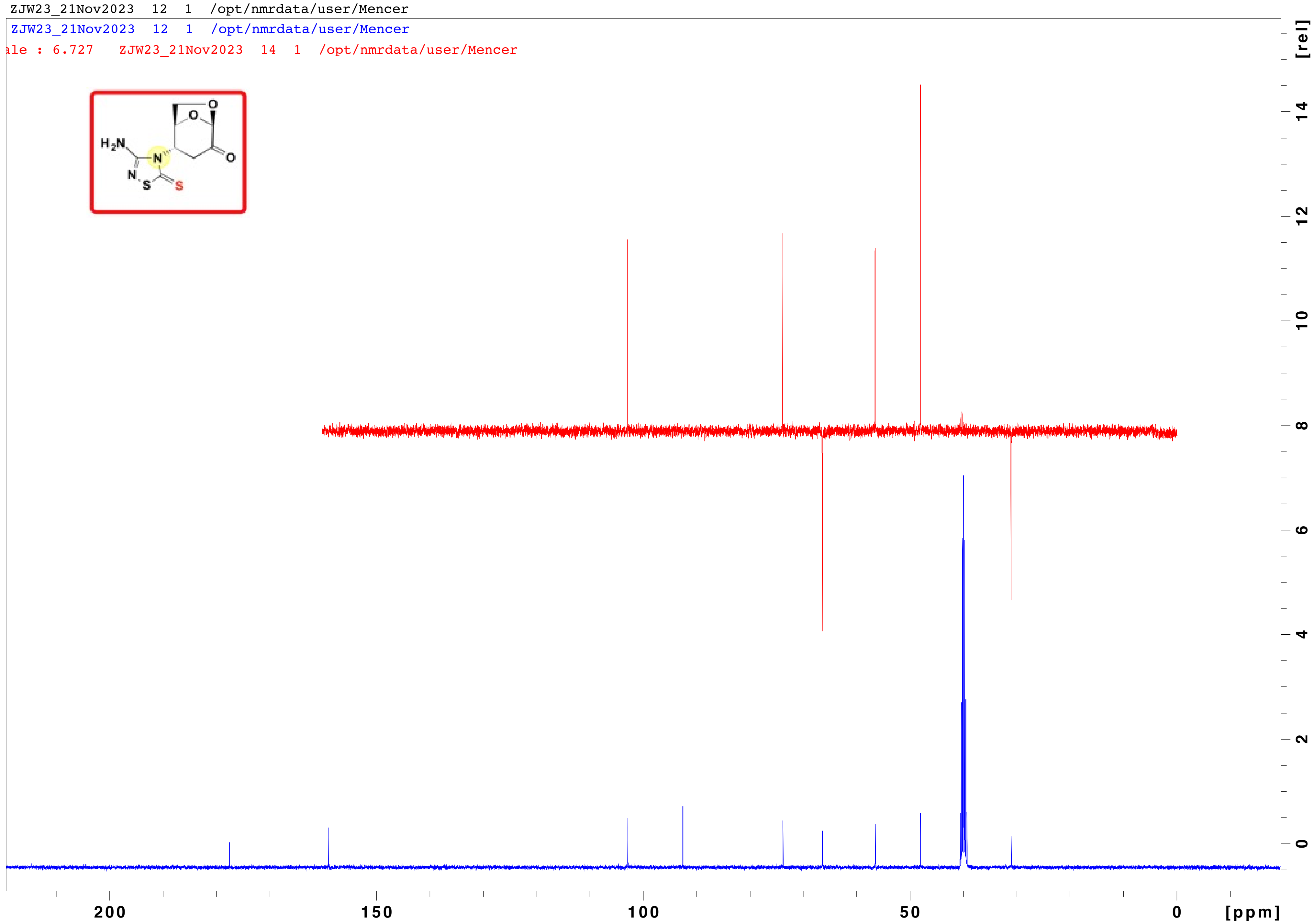

ZJW23_21Nov2023 12 1 /opt/nmrdata/user/Mencer
ZJW23_21Nov2023 12 1 /opt/nmrdata/user/Mencer
ale : 6.727 ZJW23_21Nov2023 14 1 /opt/nmrdata/user/Mencer
H2N
N
N
S
S
O
O
O
[rel]
14
12
10
8
6
4
2
0
200
150
100
50
0
[ppm]

ZJW-23 ~18 mg in ~0.67 mL DMSO
hemiketal

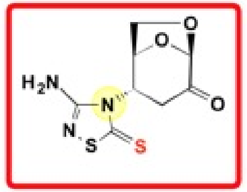

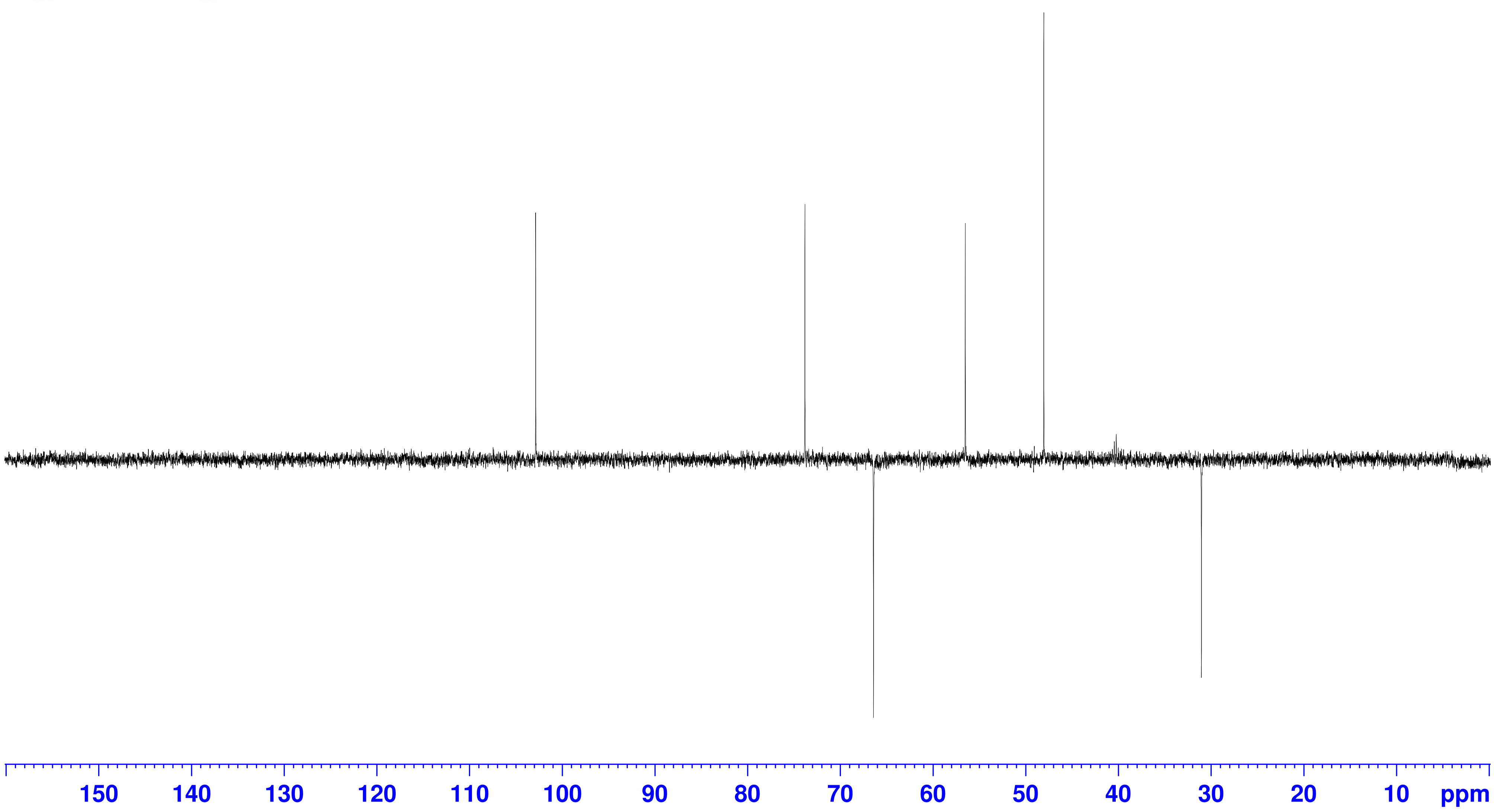

ZJW23_21Nov2023 11 1 /opt/nmrdata/user/Mencer

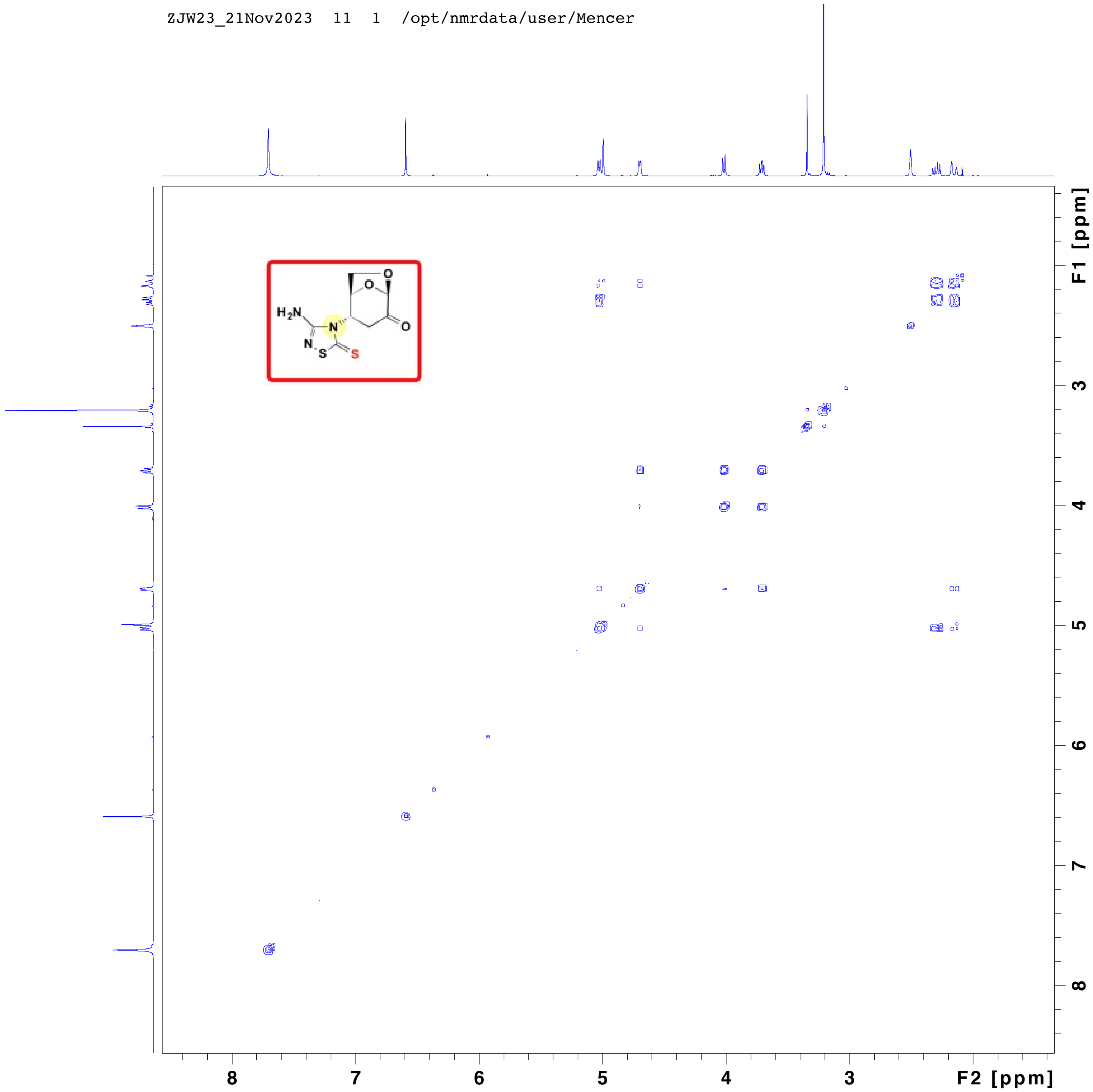

ZJW23_21Nov2023 13 1 /opt/nmrdata/user/Mencer

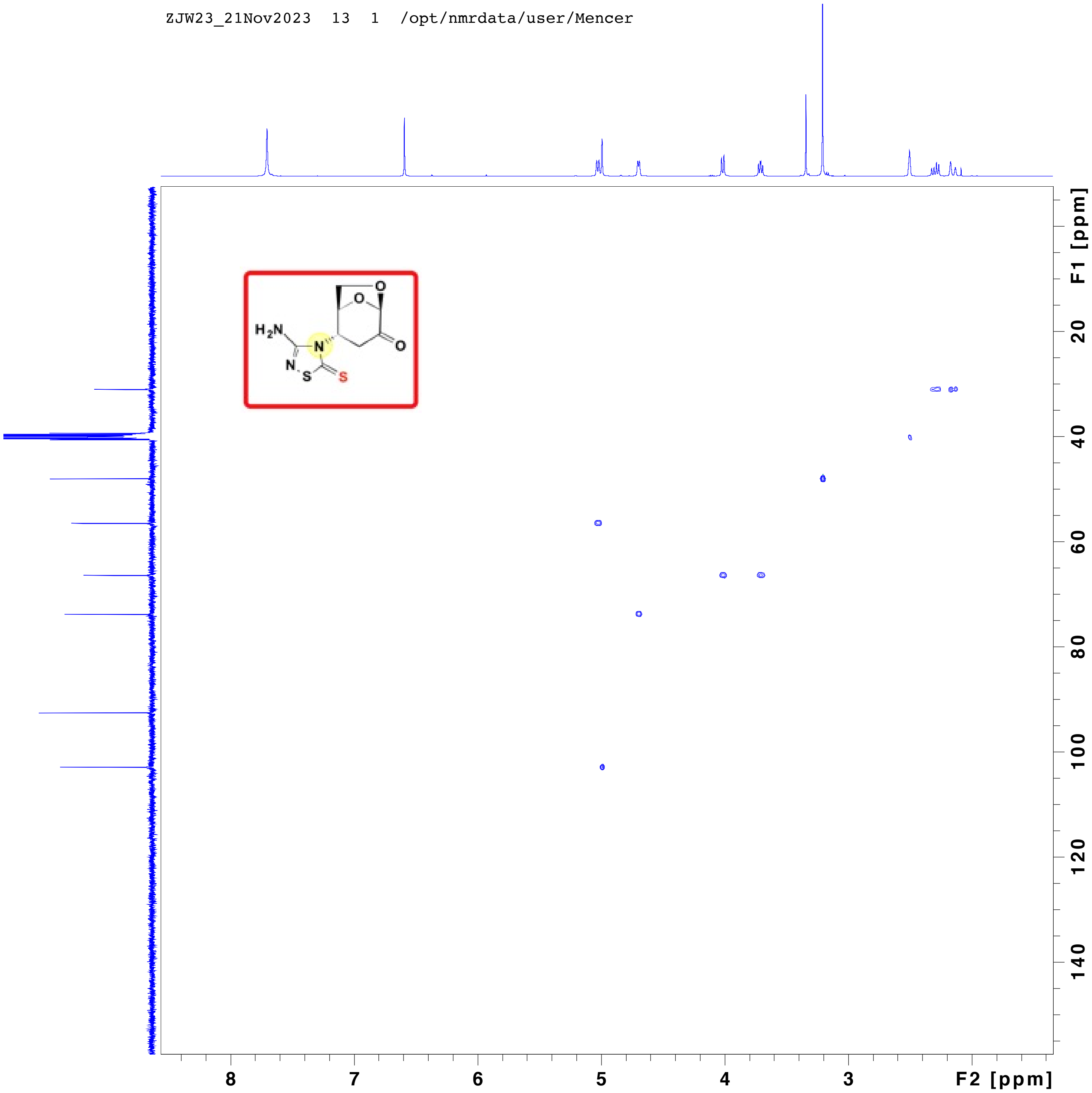

ZJW-26 ~19 mg in ~0.67 mL CDCl3

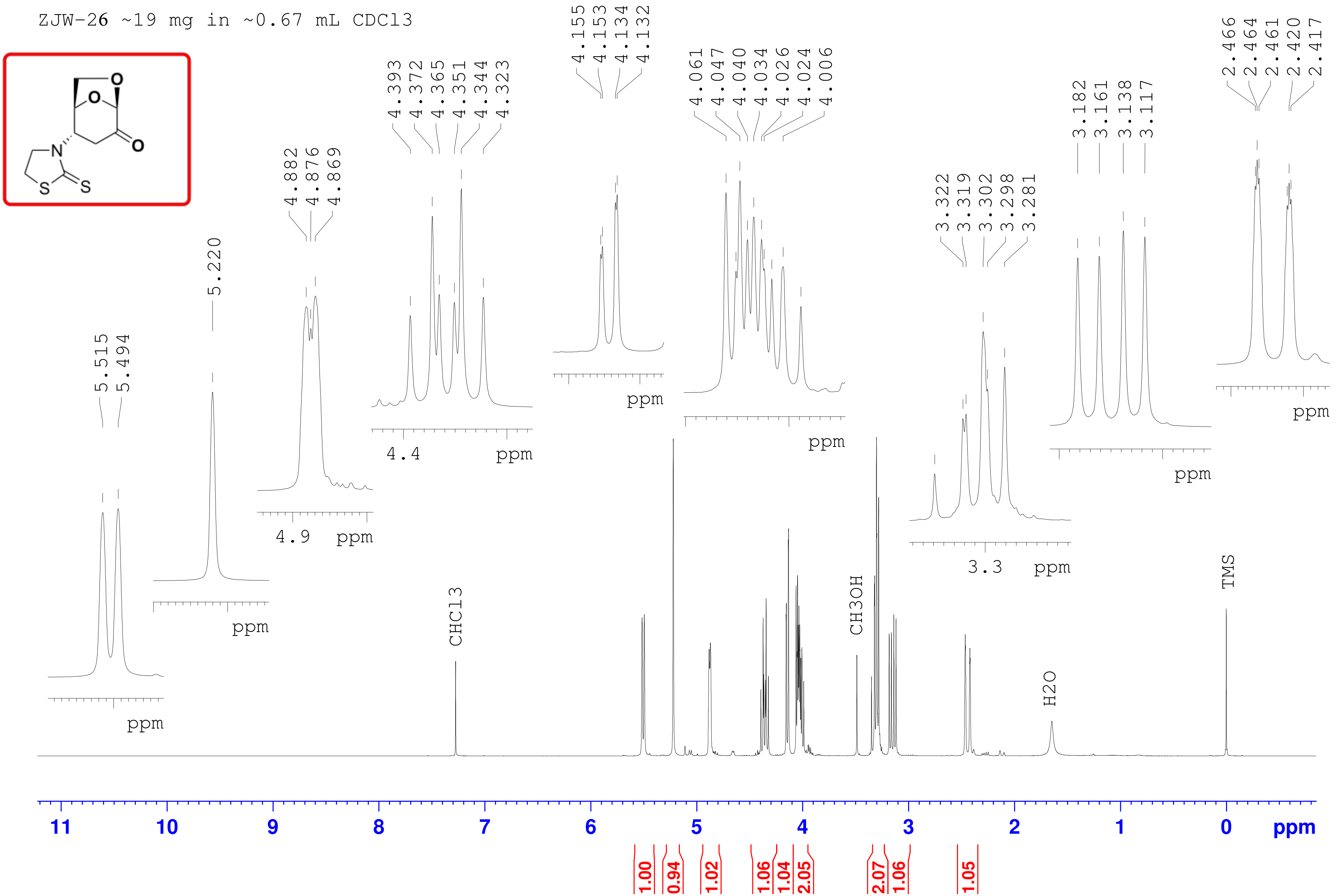

ZJW-26 ~19 mg in ~0.67 mL CDCl3

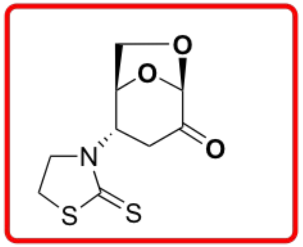

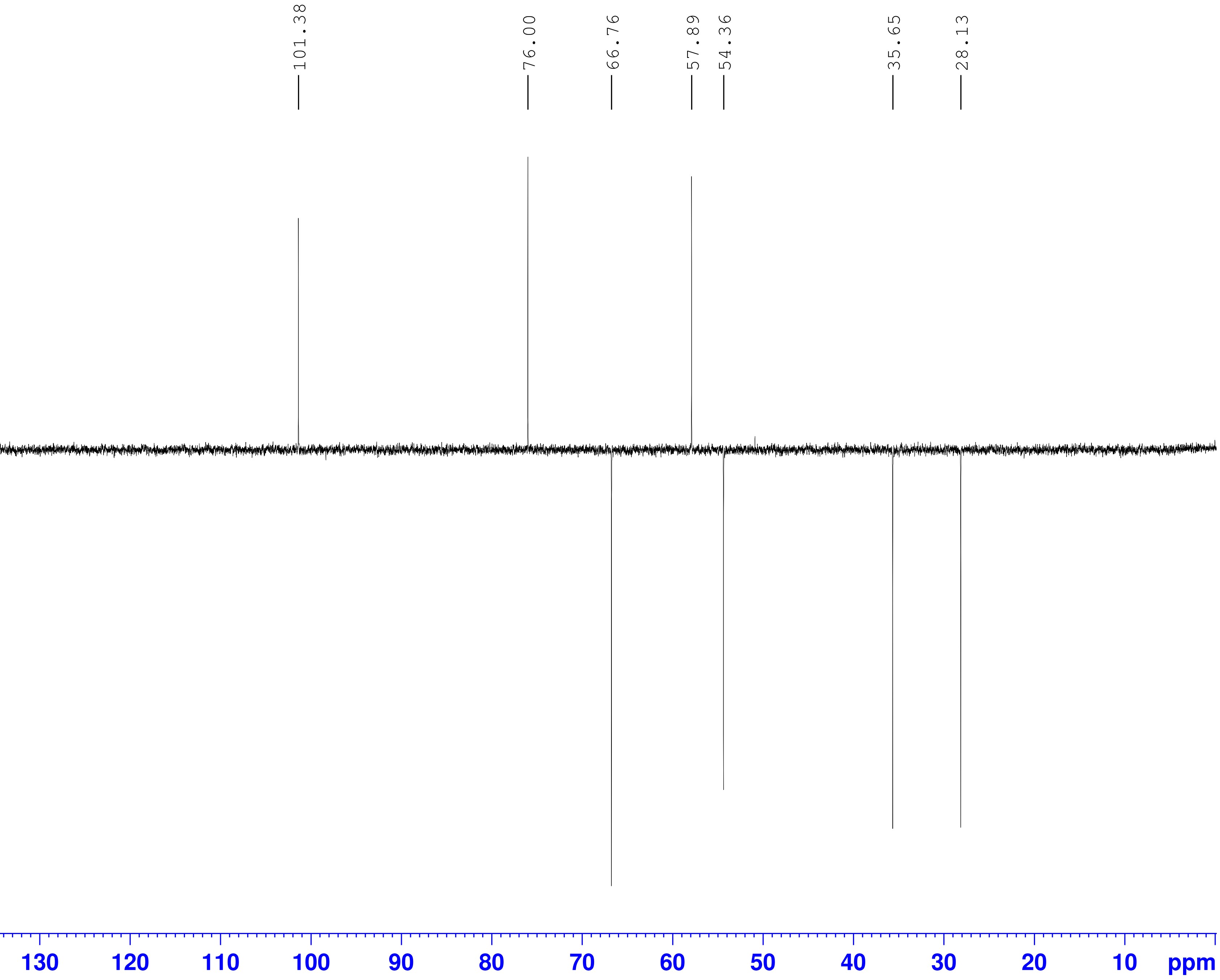

ZJW-26 ~19 mg in ~0.67 mL CDCl3

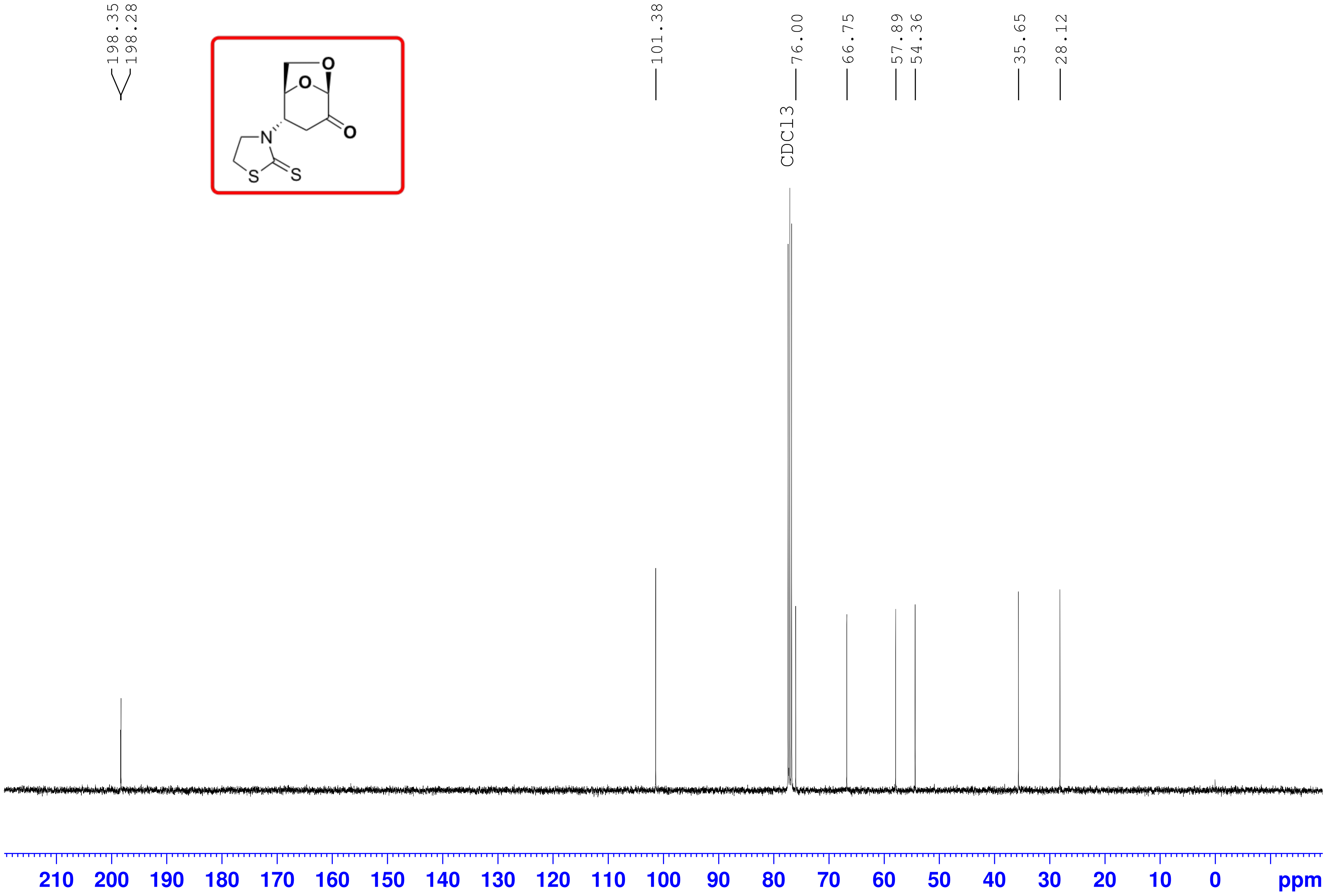

ZJW-26 ~19 mg in ~0.67 mL CDCl3

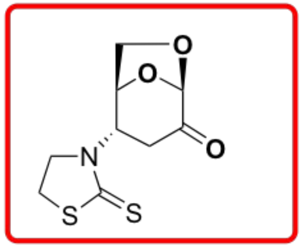

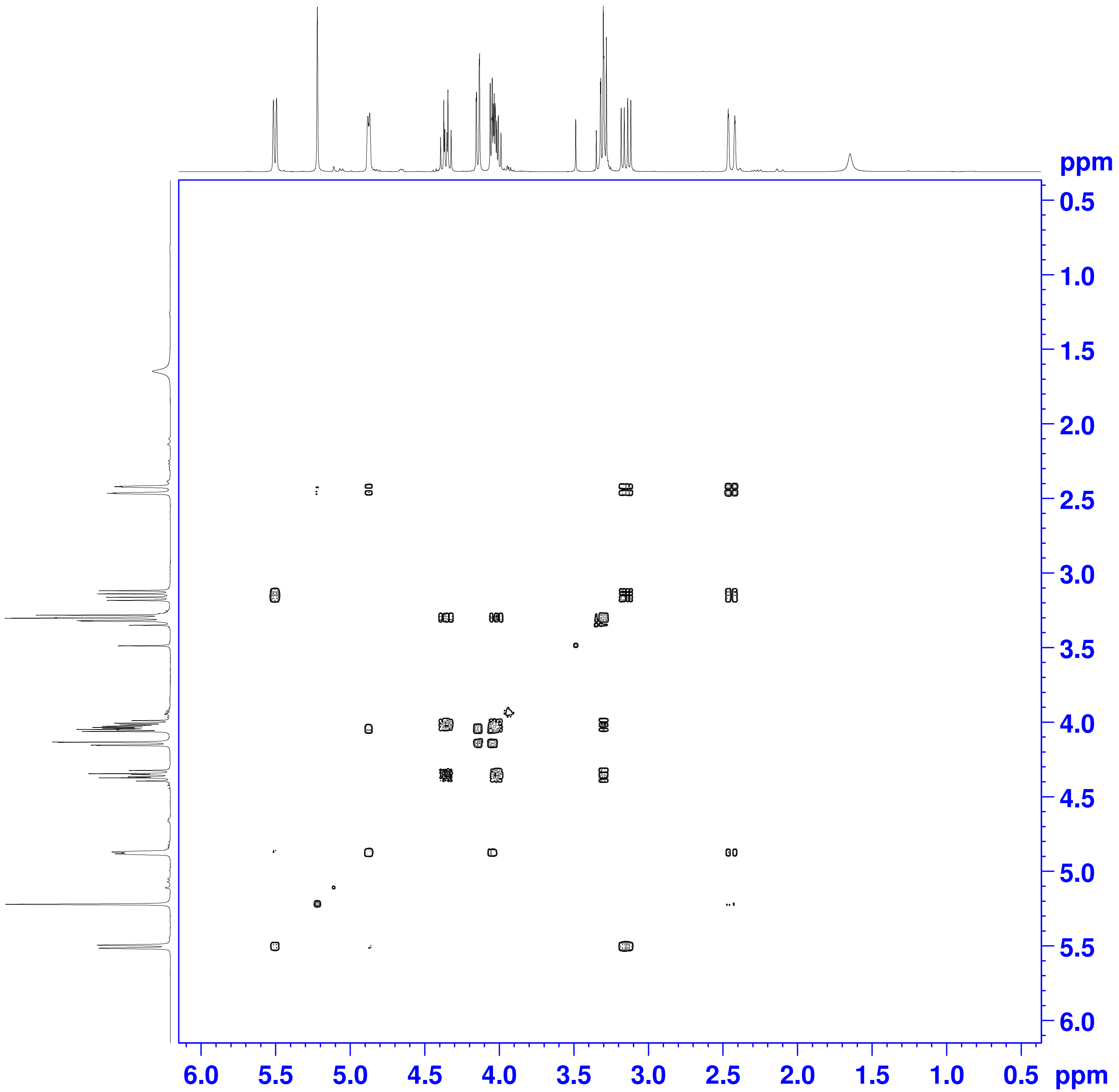

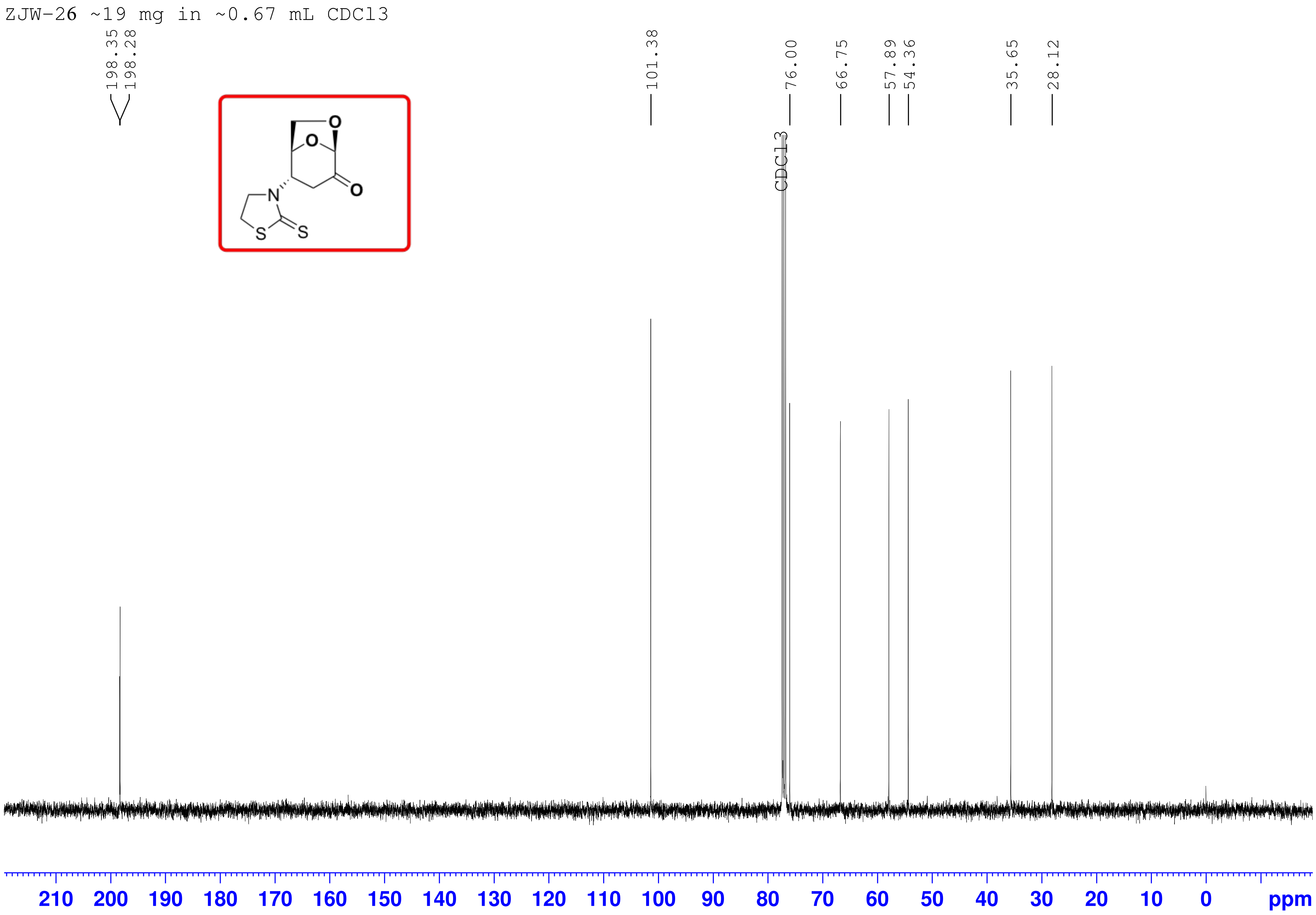
ZJW-26 ~19 mg in ~0.67 mL CDCl3
198.35
198.28
101.38
76.00
66.75
57.89
54.36
35.65
28.12
CDCl3
O
O
N
O
S
S
210 200 190 180 170 160 150 140 130 120 110 100 90 80 70 60 50 40 30 20 10 0 ppm

ZJW26_20October2023   12   1   /opt/nmrdata/user/Mencer

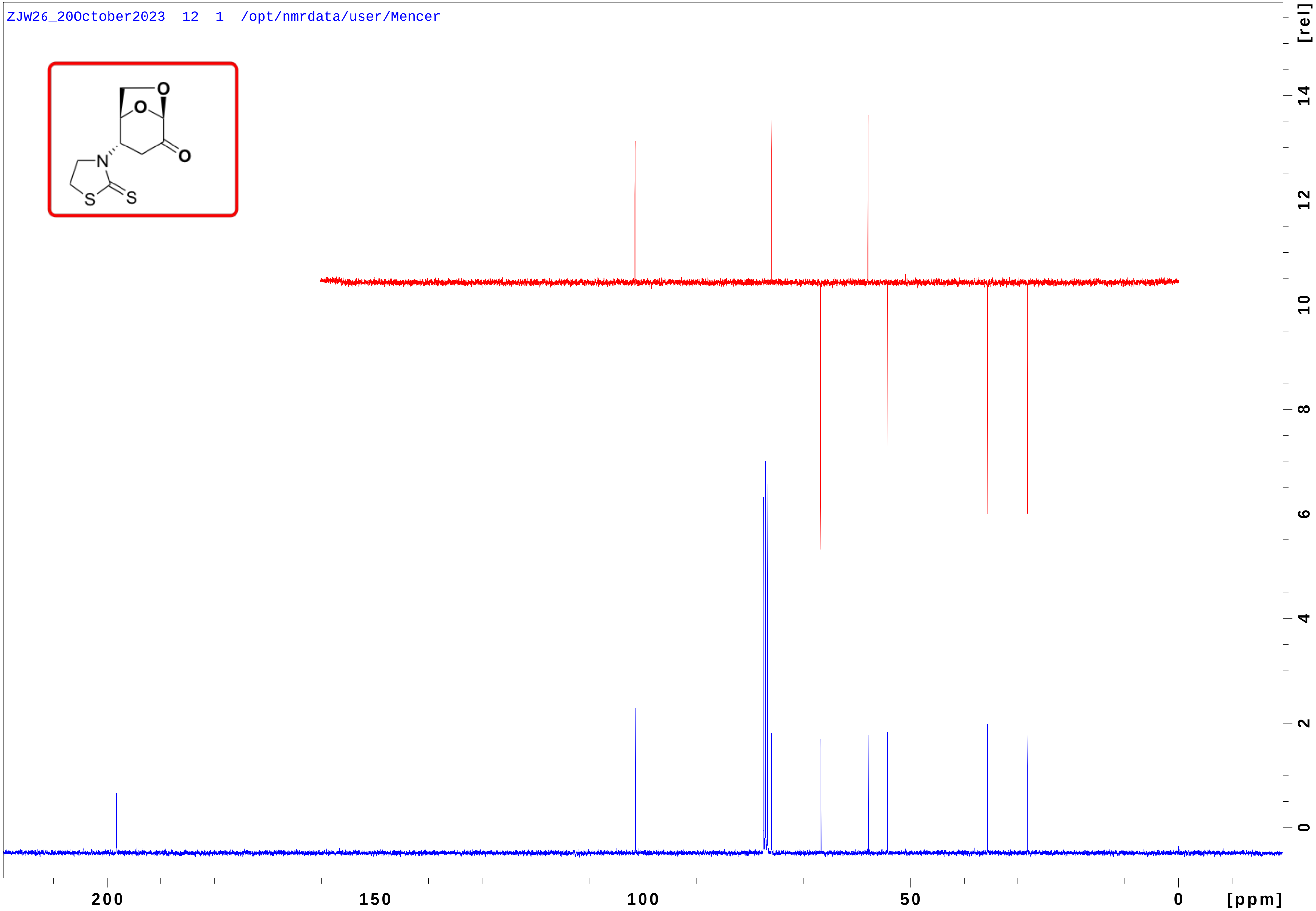

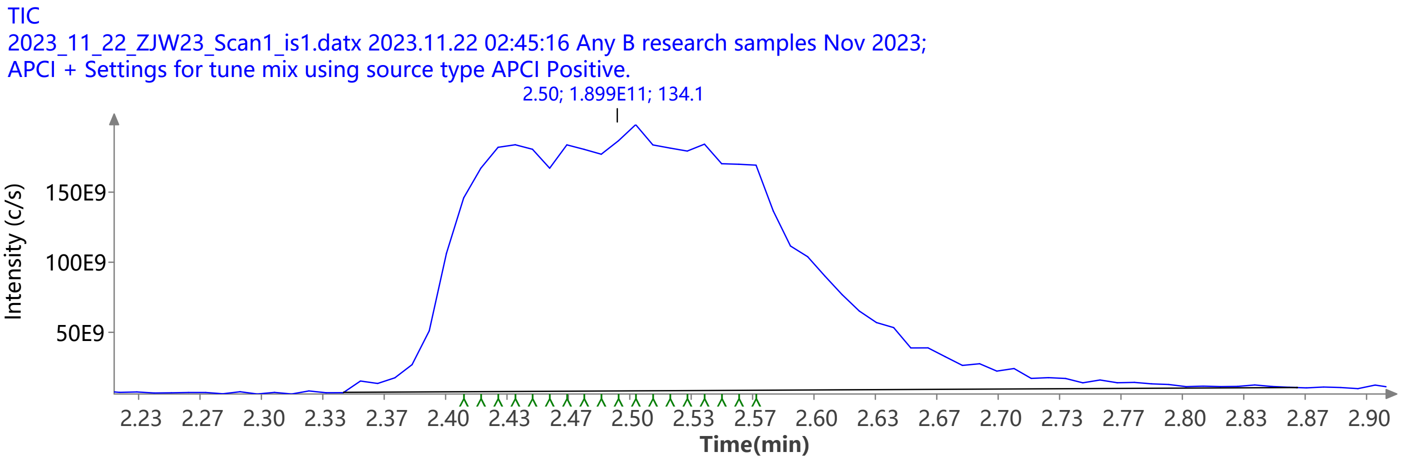

Time (Peak Maximum M:S/Minutes)	Maximum Intensity (c/s)	Time (Peak Centroid M:S/Minutes)	Peak Area	% Peak Area	Peak Resolution	Base Peak Mass (m/z)	Label
2.50	1.899E11	2.49	2.27E12	43.6	11.8	134.1	

Background RT 4.76 - 5.83 {115 scans}
2023_11_22_ZJW23_Scan1_is1.datx;
APCI + Settings for tune mix using source type APCI Positive.

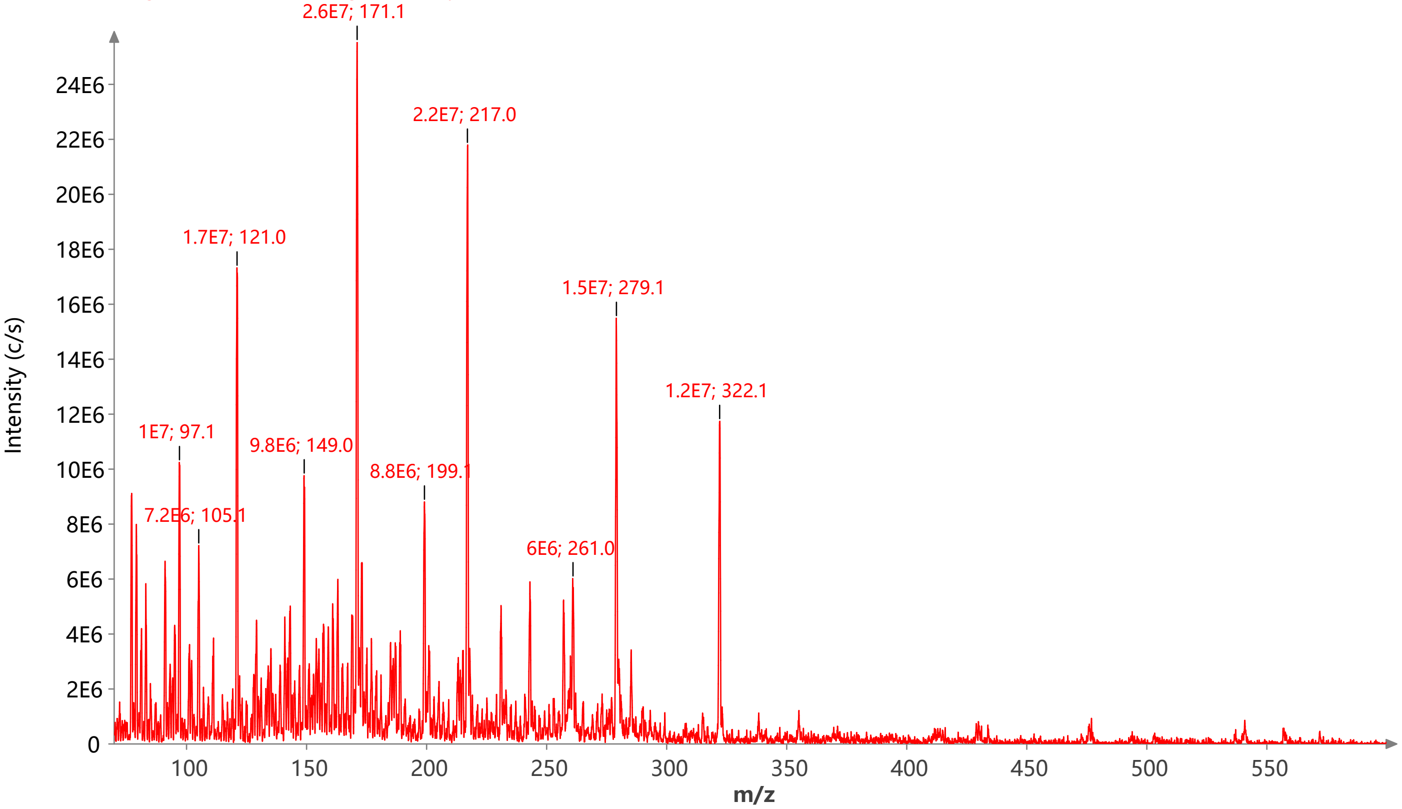

Spectrum RT 2.41 - 2.57 {18 scans} - Background Subtracted 4.76 - 5.83
2023_11_22_ZJW23_Scan1_is1.datx;
APCI + Settings for tune mix using source type APCI Positive.

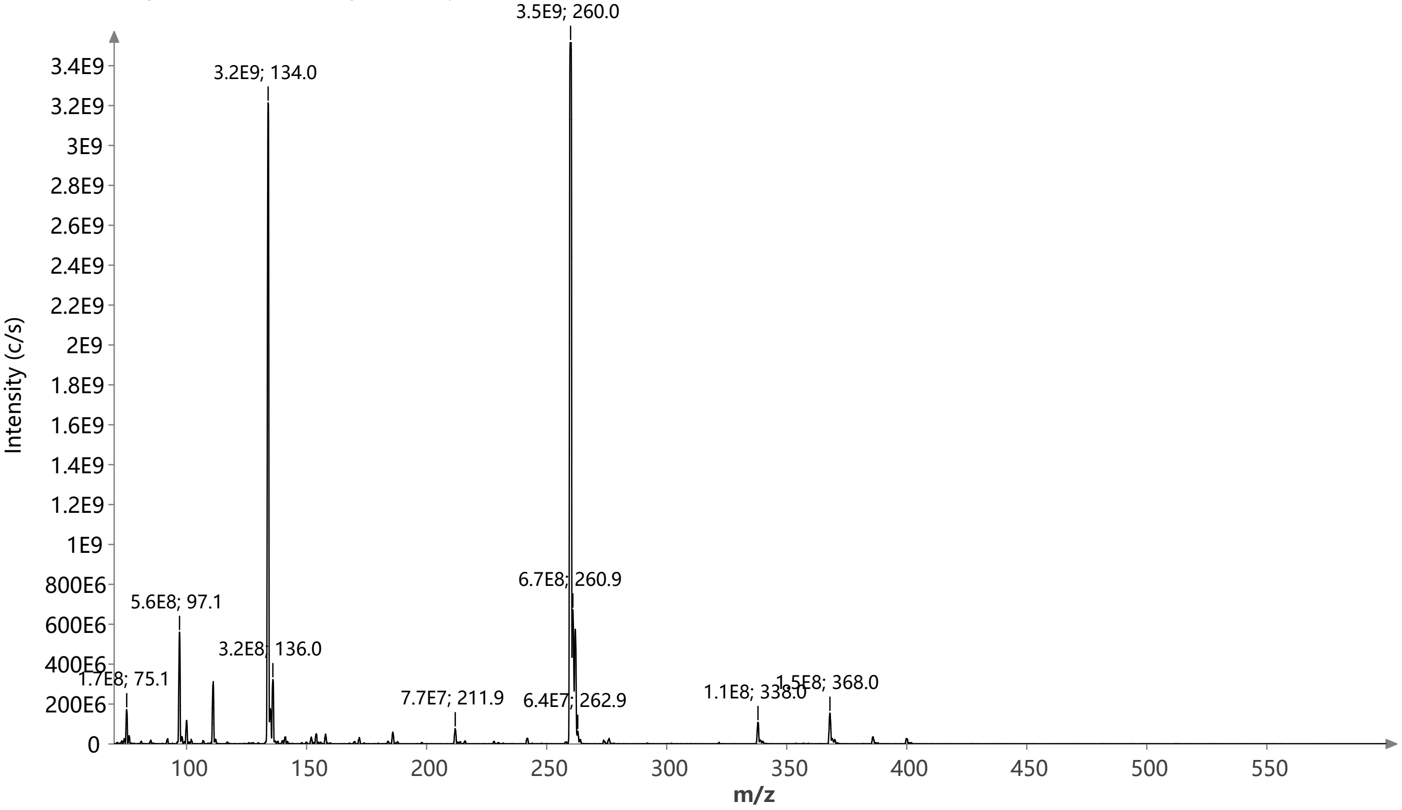

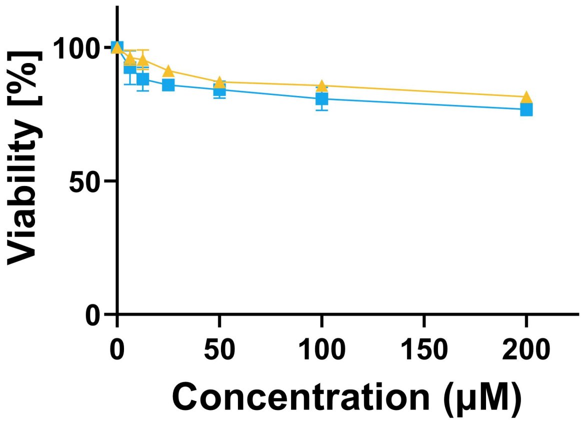

Cytotoxicity

Figure S1. The cytotoxicity of FCP23 (blue) and FCP 26 (yellow) on PTEN2 normal cells. The cytotoxicity was analyzed after 72 h incubation with FCPs in 37 °C. WST-8 is reduced by dehydrogenases in viable cells and gives a yellow colored product—formazan, which was measured with microplate reader at 450 nM. Data represent the mean ± SD.

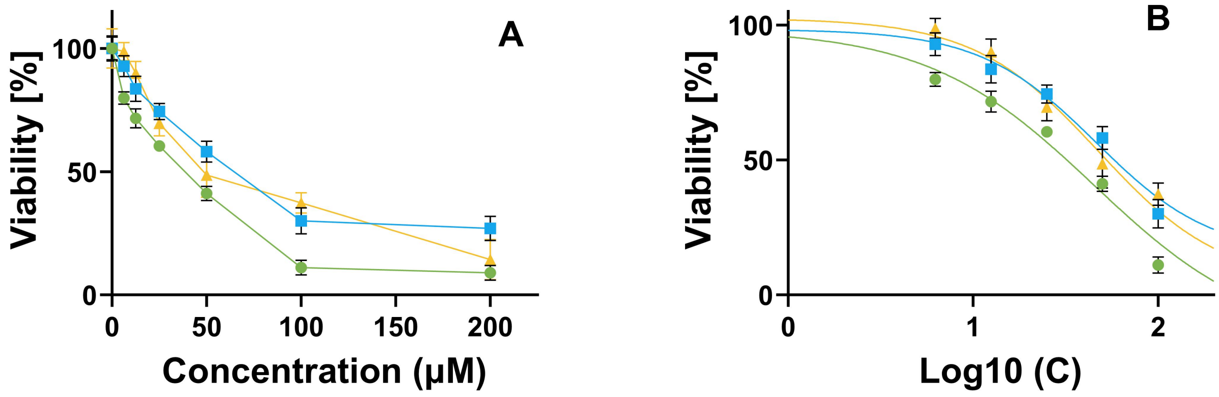

Figure S2. Representative figures demonstrating (A) the activity dependence of the varied concentrations of the etoposide (green), FCP23 (blue) and FCP 26 (yellow) along with (B) the approximation curves
